# Neurofilament light chain plasma levels are associated with area of brain damage in experimental cerebral malaria

**DOI:** 10.1038/s41598-022-14291-x

**Published:** 2022-06-24

**Authors:** Chi Ho Wai, Jessica Jin, Marek Cyrklaff, Christel Genoud, Charlotta Funaya, Julia Sattler, Aleksandra Maceski, Stephanie Meier, Sabine Heiland, Michael Lanzer, Friedrich Frischknecht, Jens Kuhle, Martin Bendszus, Angelika Hoffmann

**Affiliations:** 1 Department of Neuroradiology, Heidelberg University Hospital, Heidelberg, Germany; 2 Centre for Infectious Diseases, Parasitology Unit, Heidelberg University Hospital, Heidelberg, Germany; 3 Electron Microscopy Facility, Faculty of Biology and Medicine, University of Lausanne, Lausanne, Switzerland; 4 Electron Microscopy Core Facility, Heidelberg University, Heidelberg, Germany; 5 Neurologic Clinic and Policlinic, MS Center and Research Center for Clinical Neuroimmunology and Neuroscience Basel (RC2NB), University Hospital Basel, University of Basel, Basel, Switzerland; 6 German Center for Infection Research (DZIF), Heidelberg, Germany; 7 Department of Neuroradiology, University Institute of Diagnostic and Interventional Neuroradiology, University Hospital Bern, Inselspital, University of Bern, Freiburgstrasse, 3010 Bern, Switzerland

**Keywords:** Neuroscience, Biomarkers, Neurology

## Abstract

Neurofilament light chain (NfL), released during central nervous injury, has evolved as a powerful serum marker of disease severity in many neurological disorders, including infectious diseases. So far NfL has not been assessed in cerebral malaria in human or its rodent model experimental cerebral malaria (ECM), a disease that can lead to fatal brain edema or reversible brain edema. In this study we assessed if NfL serum levels can also grade disease severity in an ECM mouse model with reversible (n = 11) and irreversible edema (n = 10). Blood–brain-barrier disruption and brain volume were determined by magnetic resonance imaging. Neurofilament density volume as well as structural integrity were examined by electron microscopy in regions of most severe brain damage (olfactory bulb (OB), cortex and brainstem). NfL plasma levels in mice with irreversible edema (317.0 ± 45.01 pg/ml) or reversible edema (528.3 ± 125.4 pg/ml) were significantly increased compared to controls (103.4 ± 25.78 pg/ml) by three to five fold, but did not differ significantly in mice with reversible or irreversible edema. In both reversible and irreversible edema, the brain region most affected was the OB with highest level of blood–brain-barrier disruption and most pronounced decrease in neurofilament density volume, which correlated with NfL plasma levels (r = − 0.68, p = 0.045). In cortical and brainstem regions neurofilament density was only decreased in mice with irreversible edema and strongest in the brainstem. In reversible edema NfL plasma levels, MRI findings and neurofilament volume density normalized at 3 months’ follow-up. In conclusion, NfL plasma levels are elevated during ECM confirming brain damage. However, NfL plasma levels fail short on reliably indicating on the final outcomes in the acute disease stage that could be either fatal or reversible. Increased levels of plasma NfL during the acute disease stage are thus likely driven by the anatomical location of brain damage, the olfactory bulb, a region that serves as cerebral draining pathway into the nasal lymphatics.

## Introduction

Neurofilament light chain (NfL) has recently been proposed as a promising novel serum biomarker, that precisely diagnoses injury in central and peripheral nervous system^1–4^.

NfL is a subunit of neurofilaments, which are assemblies located in the neuronal cytoplasm^5^. Neurofilaments are considered neuron-specific intermediate filaments, as their diameter is about 10 nm, thus between the size of actin filaments (6 nm) and microtubules (25 nm)^6^. Neurofilaments are kept at a minimum distance from each other by side arms that project perpendicularly from the filament core^5^. A large numbers of neurofilaments has been detected and classified depending on size, type and the location in different compartments of neurons^6,7^. Neurofilaments are predominantly localised in axons, but are also present in perikarya, and dendrites^5^.

Previously, NfL has mainly been determined in cerebrospinal fluid (CSF), where it reaches the highest concentrations, but the introduction of single-molecule array (SiMoA) assays enabled the measurement of even very low NfL levels also in clinical blood samples. A study in HIV patients was the first to measure NfL levels with SiMoA and showed that CSF and plasma concentrations were highly correlated^8^. Not only in HIV, but also in other infectious diseases such as varicella zoster virus central nervous system infection, severely ill COVID-19 patients or experimental pneumococcal meningitis elevated blood NfL levels can be detected^9–11^.

In recent years NfL has evolved as a powerful marker of disease severity in neurological disorders, especially in multiple sclerosis and neurodegenerative diseases such as Alzheimer’s disease, but also in acute neurological diseases such as ischemic stroke or traumatic brain injury^1–4^.

So far, NfL has not been assessed in cerebral malaria (CM), a severe complication of the *Plasmodium falciparum* infection which is defined by unarousable coma leading to a wide range of neurological sequelae in pediatric patients^12^. One hallmark of the acute stage of CM is cerebral brain swelling, that can be detected non-invasively by magnetic resonance imaging (MRI)^13–15^. The degree of brain swelling has been identified as important predictor of disease outcome^13^. As MRI scanners are infrequently present in malaria endemic countries, blood biomarkers would have the potential to be more widely applicable.

NfL levels in cerebral malaria may thus inform about the degree of brain damage and have importance for longer term prognosis.

In order to assess if NfL levels correlate with disease severity in CM we measured plasma NfL levels in an experimental model of CM, used magnetic resonance imaging to assess disease severity, compared NfL plasma levels in a severe disease model of non-surviving mice to a newly developed disease model of moderately sick surviving mice and, assessed neurofilament ultrastructure.

## Results

### NfL plasma levels are elevated in acute disease, but do not distinguish irreversible from reversible edema in experimental cerebral malaria

To determine if NfL plasma levels could serve as a biomarker to grade edema severity in experimental cerebral malaria, we used the established ECM model with *Pb* ANKA inducing irreversible and fatal edema as well as a newly developed milder ECM model that reliably induces reversible, non-fatal edema^16^. In this study mice suffering from an irreversible edema also show a lower RMCBS (mean 4.6 ± 1.7 SD vs. mean 19 ± 1.4 SD for mice with a reversible edema) and a higher parasitemia (mean 2.1% ± 0.81 SD vs mean 1.0% 0.43 SD for mice with a reversible edema) on the day of brain extraction indicating a more severe stage of disease.

**Figure 1:**
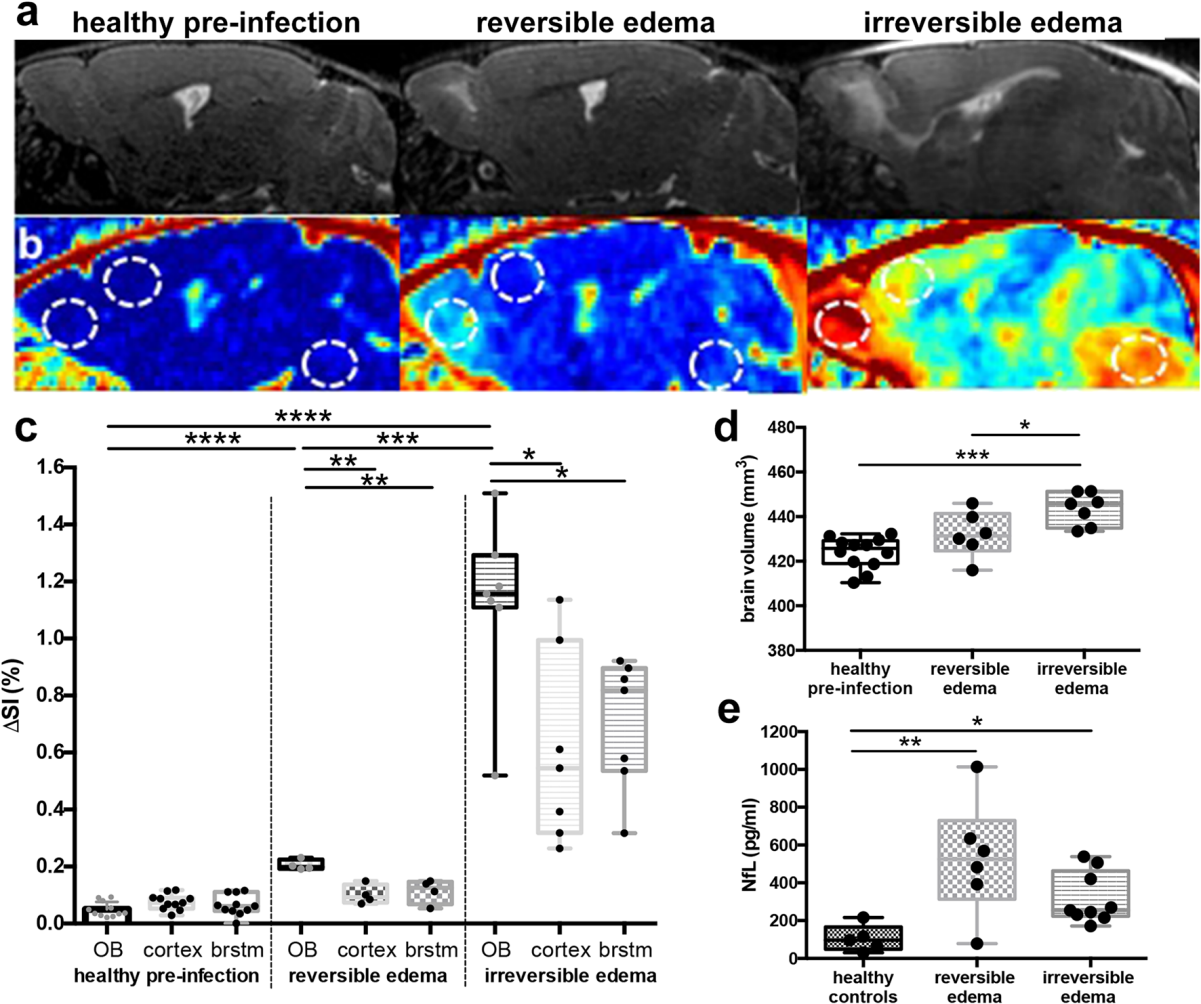
Magnetic resonance findings in experimental cerebral malaria (ECM) and neurofilament light (NfL) chain plasma levels. (**a**) Representative sagittal T2-weighted images of healthy and ECM mice with reversible and irreversible edema are shown. Healthy mice pre-infection show no pathological T2w-weighted signal increase (first image) while mice with reversible edema exhibit increased T2w signal in the olfactory bulb (second image). In contrast, mice with irreversible edema show a more pronounced edema, that extends from the olfactory bulb to the rostral and dorsal migratory stream with milder T2w signal increase also in the cortex and brainstem (third image). (**b**) T1-substraction images, of the same mice depicted in (**a**) are presented, displaying contrast agent leakage indicative of blood–brain barrier disruption (BBBD). The dotted circles highlight the regions olfactory bulb (left), cortex (middle) and brainstem (right) for each mouse. Signal intensities for these regions are plotted in (**c**). Mice with reversible and irreversible edema show a significantly higher contrast agent leakage in the olfactory bulb than healthy mice pre-infection. Mice suffering from irreversible edema show a more pronounced BBBD than healthy mice pre-infection or mice with reversible edema in all brain regions. (n_healthy pre-infection_ = 11, n_reversible edema_ = 4, n_irreversible edema_ = 7) (**d**) Total brain volumes for each mouse is displayed. Mice with more severe disease show a significantly higher brain volume compared to healthy mice pre-infection and mice with reversible edema. (n_healthy pre-infection_ = 13, n_reversible edema_ = 6, n_irreversible edema_ = 7) (**e**) NfL plasma levels are significantly increased in ECM mice compared to healthy control mice. No significant difference between reversible and irreversible edema is evident. (n_healthy controls_ = 5, n_reversible edema_ = 6, n_irreversible edema_ = 9).

As previously shown mice with irreversible edema show a higher degree of T2-weighted signal increase compared to mice with reversible edema (Fig. 1A)^16^. The olfactory bulb (OB) is the anatomical mouse brain region most affected in experimental cerebral malaria, showing earliest signs of the disease. Also blood–brain barrier disruption (BBBD) was strongest in the olfactory bulb, and the highest in irreversible fatal edema (Fig. 1B,C). While in irreversible fatal edema the cortex and brainstem also showed increased BBBD, in reversible non-fatal edema cortex and brainstem BBBD was comparable to uninfected healthy controls (Fig. 1B,C). In line with BBBD total brain volume was significantly higher in irreversible edema compared to reversible edema and healthy controls (Fig. 1D). Vessel rarefication and reduction of vascular lumen was detected by means of high-resolution MR-ToF-angiography in irreversible, but not reversible edema (Supplemental Fig. 1).

NfL plasma markers in mice with both irreversible edema (317.0 ± 45.01 pg/ml) and reversible edema (528.3 ± 125.4 pg/ml) were significantly higher compared to healthy controls (103.4 ± 25.78 pg/ml). However, despite the clear differences detected by MRI, the NfL marker did not differ significantly between irreversible and reversible edema (Fig. 1E), even showing a trend towards higher values in reversible compared to irreversible edema.

### The olfactory bulb shows highest neurofilament alterations and is associated with highest NfL plasma levels

**Figure 2:**
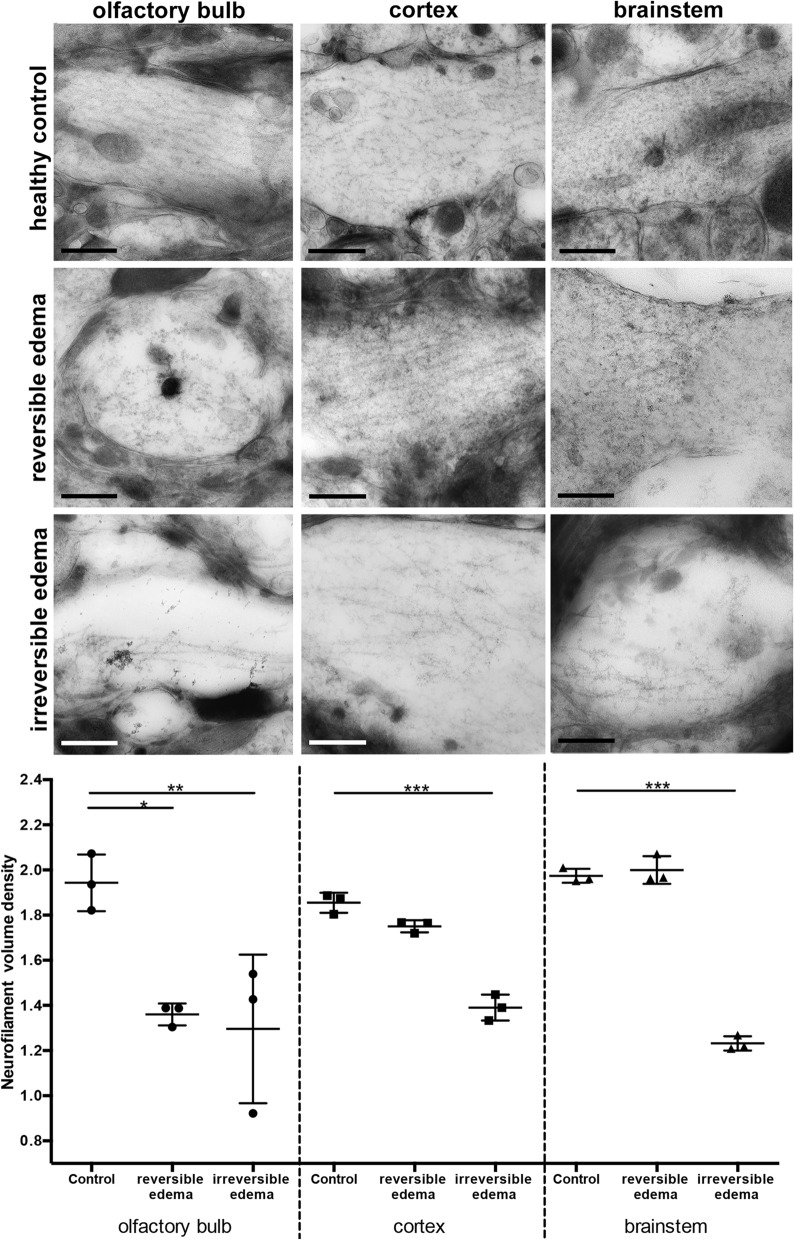
Neurofilaments in different anatomical regions in the acute disease stage. Neurofilament volume density varies depending on disease severity and brain region. EM images show representative neuronal processes for the brain regions olfactory bulb (OB), cortex and brainstem, each in healthy controls, mice with reversible edema and irreversible edema. In control mice neurofilaments run in parallel, while there is a clear loss of neurofilament density and network organization in the OB in mice with reversible and irreversible edema. While no neurofilament alterations are seen in the cortex and brainstem of mice with reversible edema, they are altered in the cortex and brainstem of mice with irreversible edema. The histograms in the last row summarize the measurements of the neurofilaments volume density for each analyzed area of the brain in progressions of disease and in control. Scale bar 200 nm. (n_healthy controls_ = 3 per region, n_reversible edema_ = 3 per region, n_irreversible edema_ = 3 per region).

To correlate the measured NfL plasma values with neurofilament alterations within the brain, we ultrastructurally assessed neurofilaments in the OB, cortex and brainstem, which were the brain areas most affected during the disease as identified by MRI. Neurofilaments were identified by their shape and size, and immunogold labelling of NfL in selected samples. When comparing the neurofilament volume density in the olfactory bulb, mice with reversible and irreversible edema showed a significantly lower volume density compared to the control group (p = 0.011, p = 0.006 respectively, Fig. 2). In cortex and brainstem, only mice with irreversible edema showed a significant decrease in neurofilament volume density compared to the control group (p < 0.001), with lowest neurofilament volume density in the brainstem. Mice with reversible edema did not show a significantly different neurofilament volume density in cortex and brainstem when compared to the olfactory bulb. Likewise, cortex and brainstem in mice with reversible edema did not differ significantly in terms of neurofilament volume density in comparison to the control group (Fig. 2).

### Neurofilament volume density in the olfactory bulb negatively correlates with NfL plasma levels

**Figure 3:**
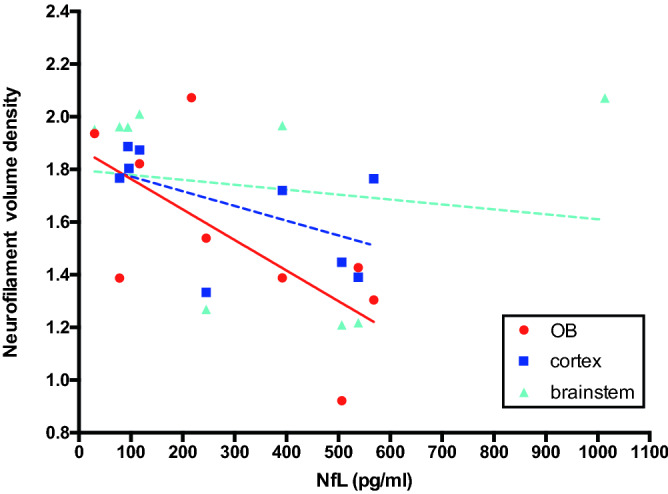
Neurofilament density volume in the olfactory bulb negatively correlates with serum neurofilament light chain (NfL) levels in contrast to the other regions. This suggests that changes in NfL serum levels in experimental cerebral malaria (ECM) are mainly driven by the location of brain injury and reflect less brain injuries as a sum. (n_healthy control_ = 3 per region, n_reversible edema_ = 3 per region, n_irreversible edema_ = 3 per region).

To assess if NfL plasma levels are associated with specific brain regions, we correlated NfL plasma levels with neurofilament volume density measurements. Increasing NfL plasma levels were strongly correlated with the decrease in neurofilament volume density in the OB (r = − 0.68, p = 0.045). However, they showed only negligible correlation with neurofilament volume density in the cortex or brainstem (Fig. 3).

### Neurofilaments regenerate after cure of the disease

**Figure 4:**
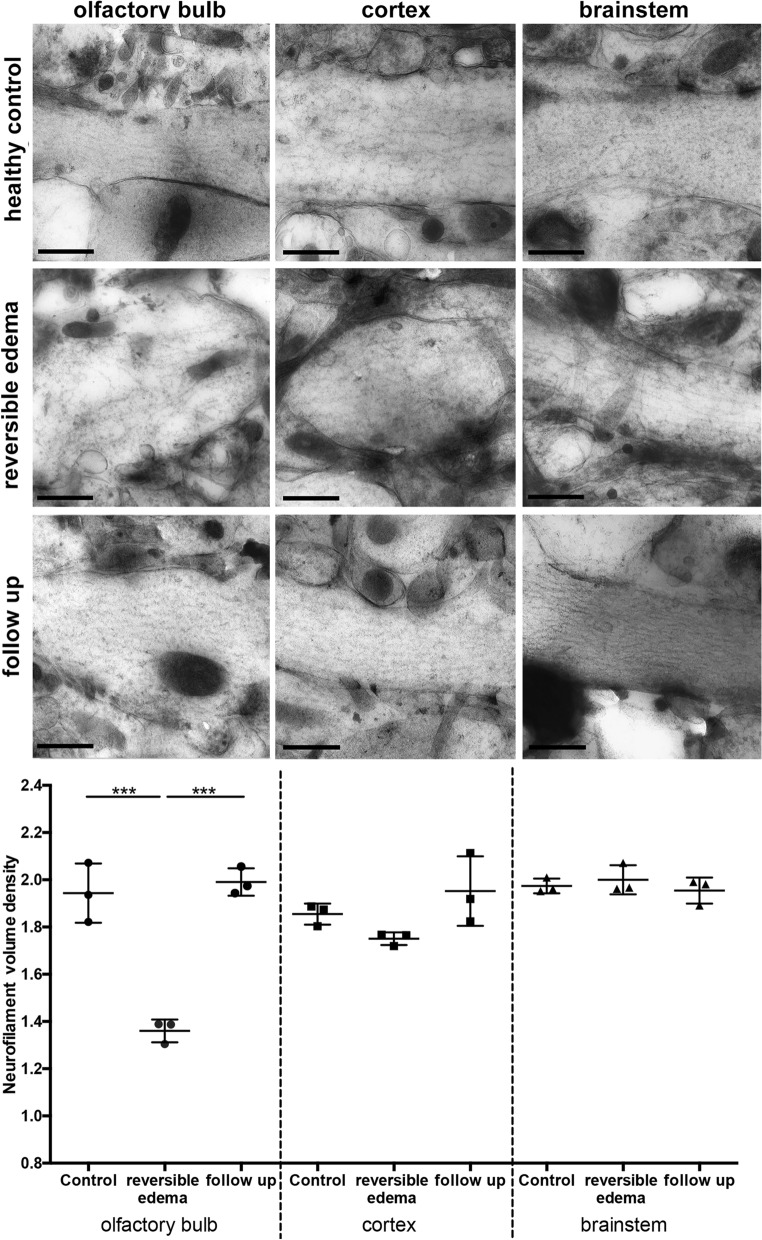
Neurofilaments in different anatomical regions at 3 months follow-up. EM images show representative neuronal processes for the following brain regions: olfactory bulb (OB), cortex and brainstem, each in healthy control, during reversible edema and in the follow-up three months after the edema. Mice show a regeneration of neurofilament density in the follow-up three months after infection. Moreover, the morphology of neurofilament networks reverses to an intact state similar to the control group. Scalebar equals 200 nm. (n_healthy control_ = 3 per region, n_reversible edema_ = 3 per region, n_reversible edema chloroquin_ = 3 per region).

To analyze long term alteration, mice that were cured of ECM underwent a follow-up examination three months after infection. Cured mice showed no pathological alterations under MRI. Visually, a regenerated neurofilament network after the cure of the disease was apparent (Fig. 4). Neurofilament volume density had normalized in the OB showing similar values to healthy control mice. Also, in cortical and brainstem regions neurofilament volume density was in the same range as healthy controls (Fig. 4). In line with those findings NfL plasma levels were in the control range 6 weeks and 5 months after infection (Supplemental Fig. 2).

## Discussion

Neurofilament Light Chain (NfL) is evolving as a powerful biomarker for various neurological diseases^1–4^. To the best of our knowledge, this study is the first to comprehensively analyze neurofilaments and its subunit neurofilament light chain by combining magnetic resonance imaging of the brain and plasma analysis with an extensive analysis on the ultrastructural level contributing to the understanding and interpretation of plasma levels of this biomarker and its relation to the area of brain damage.

We examined NfL plasma levels for the first time in ECM and observed significantly higher NfL levels during acute disease compared to controls. Also levels of plasma NfL were very high in both irreversible and reversible edema, in contrast to slower progressing diseases, such as multiple sclerosis or Alzheimer’s disease. Similar high NfL levels were observed in a mouse model of pneumococcal meningitis^11^, indicating that acute infectious diseases of the brain, such as cerebral malaria and bacterial meningitis are associated with high axonal injury. This is in line with previous studies demonstrating axonal injury in e.g. bacterial meningitis or cerebral malaria^17,18^. However, NfL levels did not reflect disease severity in this study as no significant differences of NfL levels between mice with irreversible and reversible edema in ECM were apparent. This finding contrasts previous studies in e.g. Alzheimer’s disease, multiple sclerosis or stroke that showed a strong correlation of NfL plasma levels with disease severity and/or progression^1–3^.

Disease severity in this study was measured by magnetic resonance imaging methods, namely blood–brain barrier disruption, brain edema and electron microscopy. Even though blood–brain barrier disruption and brain edema do not measure axonal injury directly, they are associated with brain parenchymal damage and often accompany axonal injury^19,20^.

The discrepancy, that NfL plasma levels did not reflect the degree of blood–brain disruption, brain swelling or the degree of neurofilament damage on electron microscopy, may be explained by several factors discussed below.

While in irreversible edema the whole brain was affected, reversible edema predominantly showed pathological alterations in the OB, a brain region of early disease involvement in ECM^16,21,22^. Previous studies have further reported a high accumulation of parasites, early blood–brain barrier disruption, microhemorrhages, microglial activation, CD8 T-cell recruitment and cell death in the OB during ECM^16,21–24^.

In terms of BBBD we observed the strongest BBBD in both irreversible and reversible edema in the OB compared to other brain regions, with BBBD being less pronounced in reversible edema. In terms of ultrastructural analysis both reversible and irreversible edema showed a similiar disparity in local neurofilament density loss in the OB, indicating a comparable neurofilament damage in both groups. BBBD has been related to increased NfL blood levels and likely contributes to the observed NfL plasma increase in ECM^25^, even though the exact mechanisms through which NfL or NfL degradation products reach the cerebrospinal fluid and the blood compartment are not known^26^. However, the perivascular and paravascular pathways of the brain that drain peptides and proteins to the lymphatic system and blood present conceivable routes^27^. As the OB is located right above the cribriform plate connecting the brain with nasal lymphatics^28–30^, it is an important drainage site for the brains’ glymphatic system^30,31^. As neurofilament volume density in the OB correlated with plasma NfL levels, NfL released at the OB may reach the blood circulation faster compared to other anatomical brain locations and thus drive plasma levels. The OB is a susceptible region of the mouse brain that shows early disease involvement in several diseases, e.g. in mouse models of Parkinson’s disease and Alzheimer’s disease, but also in patients with Alzheimer’s or Parkinson’s disease olfaction is impaired^32–34^. Not only in the mouse but also in humans CSF drains via the cribriform plate and nasal turbinates^35^ possibly explaining an earlier increase of NfL in diseases with damage to brain regions in close proximity to CSF drainage pathways.

A functional glymphatic system will drain more ‘protein waste’, e.g. amyloid-beta or albumin, which has a comparable molecular weight to neurofilament light chain, into the nasal lymphatics and consecutively into the blood^30,36^. A drainage dysfunction, likely to occur during severe ECM with impaired arterial flow and severe brain swelling, would explain why lower amounts of NfL reach the blood^22^. In addition, fatal ECM is a disease characterized by its fast progression leading to death within 24 to 48 h from the onset of edema or mild neurological symptoms. Thus, in rapidly evolving irreversible edema NfL will likely drain into the peripheral circulation only during a short period of time. In milder disease drainage function will be less impaired enabling ongoing release to the nasal lymphatics and consecutively into the blood. Other infectious diseases, e.g. experimental pneumococcus meningitis show a slower disease progression and likely allow NfL more time to reach the peripheral circulation^11^. Also, in traumatic brain injury or ischemic stroke NfL serum levels have been shown to gradually increase over time^4,37^. In ischemic stroke NfL serum levels correlated best with infarct volume after one week, but not one day after the event^37^ indicating a delayed NfL serum increase in acute neurological disorders.

Even though the reduced neurofilament density on the ultrastructural level is not a direct measure of the protein concentration within the brain, it indicated the degree of damage within specific brain regions. To the best of our knowledge this is the first study to correlate extensive ultrastructural analysis of neurofilaments with plasma NfL. The loss of neurofilaments and their alignment in the OB, measured as loss of neurofilament density, was striking in mice with irreversible and reversible edema. Similar alterations of cytoskeletal neurofilament organisation occur e.g. in stroke or epilepsy and have been related to hypoxia or excitotoxic dendritic swelling^38–40^, known to also occur during ECM^41^. The observed morphology is thus likely related to both an impaired cytoskeletal network, but also an increased intracellular water content due to a cellular edema^40^. In mice with irreversible edema the lowest neurofilament density was observed in the brainstem, which is in line with a prior study examining ECM with irreversible edema and illustrated the highest rate of neuronal cell death in the brainstem^42^. Mice with reversible edema showed neurofilament alterations only in the OB, which normalized over time.

Cerebral malaria can lead to permanent neurological or neurocognitive impairment in survivors^43^. In this study mice with reversible edema were less severely sick compared to a prior study as assessed with the RMCBS score^44^. Neither MR imaging, nor electron microscopy analysis, nor NfL plasma levels showed permanent pathological changes, indicating that regeneration occurs in mild disease without permanent cerebral damage or permanent cytoskeletal neurofilament loss. However, we did not assess behavioral or olfactory changes with more dedicated tests, and can thus not exclude that mild cognitive impairment remains after survived disease. Also even though the central nervous system is predominantly involved during malaria infection, we cannot rule out that part of the NfL increase is caused by peripheral central nervous damage^45^. Furthermore, we cannot rule out that parasitemia-induced anemia or peripheral organ involvement may contribute to increased NfL levels as damage to the central nervous system and neurocognitive impairment have been described in e.g. anemia, kidney or liver failure^46–48^. One further limitation of this study is that we did not assess CSF levels of NfL, which would have been potentially higher in mice with irreversible edema compared to mice with reversible edema. Comparing NfL levels in CSF and blood in future studies would help to better understand NfL dynamics in this disease and may eventually result in NfL threshold levels to grade brain involvement.

In conclusion neurofilament light chain levels are elevated during ECM, but do not reliably detect the disease severity in the acute stage of ECM, presumably due to a combination of glymphatic drainage dysfunction and a delay in its release to circulation. Moreover, increased levels of serum NfL appear to be mainly driven by the anatomical location of brain damage as observed in the olfactory bulb, a region that serves as CSF draining pathway into the nasal lymphatics.

## Methods

### Ethics statement

All animal experiments were performed according to FELASA category B and ARRIVE guidelines and approved by the local animal welfare committee (Regierungspräsidium Karlsruhe, Germany).

### Murine Malaria Model

Experimental cerebral malaria (ECM) was induced with the *Plasmodium berghei* ANKA (*Pb* ANKA) parasite in inbred female C57BL/6J mice (Janvier Labs, France). *Pb* ANKA sporozoites (SPZ) were isolated by dissection of salivary glands from female *Anopheles stephensi* mosquitoes at day 18–21 post infection. Infections of 12–14-week-old female mice were performed by intravenous (i.v.) injections of 1000 SPZ in a total volume of 100 µl sterile PBS. In the first group (n = 9) naïve mice were infected. In a second group (n = 6) the same infections were carried out in single vaccinated mice^49^. For immunization with radiation-attenuated sporozoites (RAS), SPZ were treated by exposure to 150 Gy of γ-radiation (^137^Cesium source, University Hospital Heidelberg, Germany) and were then injected into mice at a dose of 3 × 10^4^ RAS. In the third group (n = 5) single immunized mice, that showed reversible edema after infection, were treated with chloroquine in drinking water for 7 days according to a previously established protocol starting at day 14 post infection^50^. All treated mice showed no parasitemia after the treatment. Healthy female C57BL/6J mice (n = 5) served as controls. All groups are listed in Supplemental Table 1. Parasitemia was assessed starting at day 4 after infection. For behavioral evaluation, malaria-infected mice were assessed for ten parameters of cerebral symptoms according to the Rapid-Murine-Coma-and-Behavioral-Scale (RMCBS)^51^. The RMCBS testing was performed daily starting on day 6 after infection. After day 14 post infection the RMCBS score was assessed weekly.

### MRI protocol

MRI was performed on a 9.4 T small animal scanner (BioSpec 94/20 USR, Bruker Biospin GbmH, Ettlingen, Germany) using a volume resonator for transmission and a 4-channel-phased-array surface receiver coil. Anesthesia was induced per inhalation using 2% and maintained with 1–1.5% isoflurane. Mice were placed prone in fixed position monitoring body temperature and respiration. Mice were imaged before and after infection. Starting at day 6 after infection mice were screened for edema by using a 2D T2-w sequence (repetition time/ echo time (TR/TE = 2000/22 ms, slices = 12, slice thickness = 0.7 mm). Before infection and at edema occurrence a longer MR imaging protocol was acquired and included 2D T2-w imaging or 3D T2-w imaging (TR/TE = 2000/22 ms, 100 µm isotropic resolution, field of view = 20 × 10 × 12 mm^3^) as well as 3D T1-w imaging (TR/TE = 5/1.9 ms, flip angle = 8.5°, 156 µm isotropic resolution, field of view 20 × 18.7 × 18.7 mm^3^) before and after intravenous injection of 0.3 mmol/kg Gd-DTPA. In two severely sick mice of group 1 3D non-enhanced T1w images were not acquired in order to shorten the MRI protocol.

### Blood collection in mice

After the last MRI scans blood was obtained by heart puncture or from the mandibular vein in the third group at week 6 post infection with heparinized syringes and centrifuged at 2000×*g* for 10 min to obtain plasma. Plasma samples were then aliquoted (70 ml) and stored at − 80 °C until use.

### Electron microscopy of mouse tissue

Mouse brains were dissected, cut in halves and fixed in a solution of 4% PFA, 0.1% GA in 50 mM CaCo and 0.9% NaCl at 4 °C overnight. Brains were then cut into 150 µm thick slices using a Leica vibratome. From these slices round circles of 2 mm diameter were stanced out using a biopsy punch. The tissue coins were high pressure frozen in a HPM 010 (Bal-Tec, Liechtenstein) in 0.2 mm deep aluminium carriers (Engineering Office M. Wohlwend GmbH, Sennwald, Switzerland) and filled up with a cryoprotectant of 0.6 M sucrose, 30 mM trihalose in CaCo buffer. Afterwards, samples underwent freeze substitution in an EM-AFS2 (LeicaMicrosystems, Vienna, Austria) with 0.1% uranyl acetate in dry acetone, during which samples were gradually brought to room temperature. The protocol included rinsing in EtOH at − 60 °C followed by HM20 infiltration at 30%, 60%, and 100% at − 55 °C, − 50 °C, − 45 °C, and − 35 °C. Polymerization was done at − 35 °C with UV light. The samples were then cut into 350 nm thick sections using either a Leica UC6 or Leica UC7 ultramicrotome (LeicaMicrosystems, Vienna, Austria) and placed on formvar coated copper grids. Before imaging, sections were stained with 3% uranyl acetate in H_2_O for 3 min and Reynold’s lead citrate again for 3 min. All EM imaging was performed using a JEM JEOL-1400 operating at 80 kV and equipped with a TemCam F416 4 k × 4 k pixel digital camera by TVIPS (Gauting, Germany) and EM Menu software 4.0 by TVIPS (Gauting, Germany). For the stereological analysis images were taken at × 25,000 magnification, pixel size of 0.436 nm. For tomography 350 nm thick sections were placed on formvar coated slot grids. Tilt series were taken at every two degrees over a ± 60° range were recorded using a Tecnai F20 EM (FEI, Eindhoven, The Netherlands) operating at 200 kV using the SerialEM software package (Mastronarde 2005) on FEI Eagle 4 K × 4 K CCD camera at 2.24 nm pixel size. Tilted images were aligned by cross-correlation procedure. The tomograms were generated by weighted-back-projection algorithm using IMOD processing packages^52^.

### Image analysis

Image processing was undertaken in Amira 5.4 (FEI, Visualization Sciences Group).

Blood–brain barrier permeability (BBBD) was assessed by contrast-enhanced 3D gradient echo T1-w imaging. 3D non-enhanced T1w images were subtracted from contrast enhanced T1-weighted images. In pre- and post-contrast 3D T1-weighted images, Gibbs ringing was suppressed, and signal-to-noise-ratio enhanced using a 3D spatial Gaussian low-pass filter with a resulting effective isotropic resolution of 280 µm. In case of significant motion between the sequences, images were motion corrected using a custom-made MATLAB code for rigid body registration. First the difference images were evaluated for pathological enhancement by visual inspection. In 2 mice of group 2 motion correction failed and data sets of those mice were excluded from analysis. In 2 mice of group 1 difference images could not be calculated due to acquired 3D non-enhanced T1w images.

Second, the relative signal enhancement ΔSI (%) in different regions-of-interest (ROI) was quantified as: ΔSI (%) = [(SI_post contrast_ − SI_pre contrast_)/SI_pre contrast_ ] × 100%. ROIs were placed after anatomical delineation manually into the olfactory bulb, cortex and brainstem according to the Allen Brain Atlas^53^. Brain volume was semi-quantitatively assessed by using 3D non-enhanced T1w images.

### Stereological analysis

To allow a proper differentiation of neurofilaments from other components of the cytoplasm, the analysis was restricted to dendrites showing strictly transversally sectioned neurofilaments. The volume density analysis was performed with the grid tool in FIJI^54^. Crosses and horizontal lines were projected in a random offset onto the images with an area per point of 1,000,000.00 nm^2^. For the volume of the dendrites, the amount of crosses within one dendrite (*V*) was counted and served as a surrogate measure. The amount of neurofilaments within that dendrite was counted as the number of times (*n*) a neurofilament crossed the horizontal lines projected. The volume density (*D*) for one subject was then calculated by dividing the total number of times a neurofilament crossed the horizontal lines by the total amount of crosses counted within the dendrites ($D= \frac{n}{V}$). For each subject and time point at least one hundred events were counted with an event being either the surrogate measure for the volume of the dendrites or the surrogate measure for the amount of neurofilaments within the dendrites.

### Electrochemiluminescence immunoassay to measure NfL

NfL concentrations in plasma were measured in duplicates using a previously described and validated electrochemiluminescence (ECL) immunoassay (capture monoclonal antibody: 47:3; biotinylated detector monoclonal antibody: 2:1, UmanDiagnostics AB, Umeå, Sweden^55^) with some modifications^56^. Murine plasma samples were prediluted 1:2 in sample diluent. Parallelism and linearity of the assay was confirmed by serial dilution experiments^57^. All NfL measurements were performed in a blinded fashion.

### Statistics

Data are shown as mean ± standard error of mean (SEM) Statistical analyses was performed in PRISM (GraphPad, version 8, La Jolla, CA). To compare two groups, unpaired, two tailed student’s t-tests were used (e.g. parasitemia non-survivor, survivor). To compare more than two experimental groups (baseline/control, non-survivor, survivor) one-way analysis of variance (ANOVA) with multiple comparisons was performed. Spearman’s analyzes were used for correlation analyses. p values ≤ 0.05 were considered statistically significant.

## Supplementary Information

Supplementary Information.

## Data Availability

Data will be made available by the corresponding author on reasonable request.
