# Supplementary material for: Neurofilament light chain plasma levels are associated with area of brain damage in experimental cerebral malaria
Source: Sci Rep. 2022 Jun 24;12:10726. doi: 10.1038/s41598-022-14291-x (PMC9232608; doi:10.1038/s41598-022-14291-x)
Supplement: Supplementary file 1 — Supplementary Information. [file 41598_2022_14291_MOESM1_ESM.pdf]

Supplemental Material

Neurofilament light chain plasma levels are associated with area of brain damage in experimental cerebral malaria

Chi Ho Wai,[1,2] Jessica Jin,[1,2] Marek Cyrklaff,[2] Christel Genoud,[3] Charlotta Funaya,[4] Julia Sattler,[2] Aleksandra Maceski,[5] Stephanie Meier,[5] Sabine Heiland,[1] Michael Lanzer,[2] Friedrich Frischknecht,[2,6] Jens Kuhle,[5] Martin Bendszus,[1] Angelika Hoffmann[1,7*]

[1] Department of Neuroradiology, Heidelberg University Hospital, Germany
[2] Centre for Infectious Diseases, Parasitology Unit, Heidelberg University Hospital, Heidelberg, Germany
[3] Electron Microscopy Facility, Faculty of Biology and Medicine, University of Lausanne, Switzerland.
[4] Electron Microscopy Core Facility, Heidelberg University, Heidelberg, Germany
[5] Neurologic Clinic and Policlinic, MS Center and Research Center for Clinical Neuroimmunology and Neuroscience Basel (RC2NB), University Hospital Basel, University of Basel, Basel, Switzerland
[6] German Center for Infection Research (DZIF), Heidelberg, Germany
[7] University Institute of Diagnostic and Interventional Neuroradiology, University Hospital Bern, Inselspital, University of Bern, Switzerland

Supplemental Table 1: Experimental groups. The four experimental groups are listed. '1' indicated that MRI, NfL measurements, brain volume (brain vol) measurements, BBBD measurements, EM analysis was performed. '0' indicated that the analysis was not performed.

group_number	MRI	NfL	brain vol	BBBD	EM	EM OB	EM Ctx	EM brstm
Control 1	1	1	0	0	1	0	1	1
Control 2	1	1	0	0	1	1	0	1
Control 3	1	1	0	0	1	1	1	1
Control 4	1	1	0	0	1	0	1	0
Control 5	1	1	0	0	1	1	0	0
irreversible-edema_1	1	1	1	1	1	1	1	1
irreversible-edema_2	1	1	1	1	1	1	1	1
irreversible-edema_3	1	1	1	1	1	1	1	1
irreversible-edema_4	1	1	1	1	0	0	0	0
irreversible-edema_5	1	1	1	1	0	0	0	0
irreversible-edema_6	1	1	1	1	0	0	0	0
irreversible-edema_7	1	1	1	1	0	0	0	0
irreversible-edema_8	1	1	0	0	0	0	0	0
irreversible-edema_9	1	1	0	0	0	0	0	0
reversible-edema_1	1	1	1	1	1	1	1	1
reversible-edema_2	1	1	1	1	1	1	1	0
reversible-edema_3	1	1	1	1	1	1	1	1
reversible-edema_4	1	1	1	1	1	0	0	1
reversible-edema_5	1	1	1	0	0	0	0	0
reversible-edema_6	1	1	1	0	0	0	0	0
reversible-ed-chloroquin_1	1	1	0	0	1	1	1	1
reversible-ed-chloroquin_2	1	1	0	0	1	1	1	1
reversible-ed-chloroquin_3	1	1	0	0	1	1	1	1
reversible-ed-chloroquin_4	1	1	0	0	0	0	0	0
reversible-ed-chloroquin_5	1	1	0	0	0	0	0	0
total (n)	25	25	13	11	15	12	12	12

Supplemental Figures

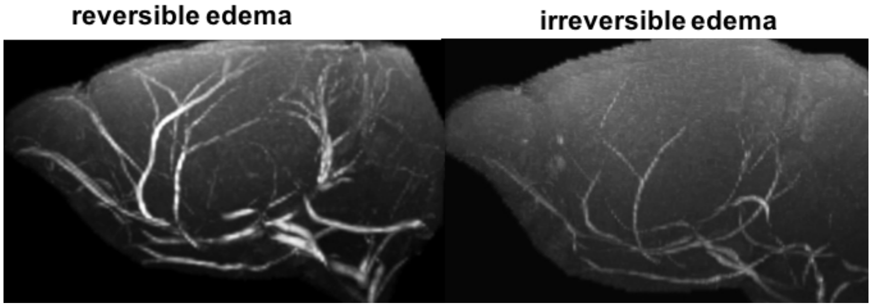

Supplemental Figure 1: Arterial vessel rarefication in irreversible versus reversible edema
MIP-reconstructed TOF angiographies of mice with reversible and irreversible edema are shown. Mice with irreversible edema show vessel rarefication indicating diminished cerebral blood flow.

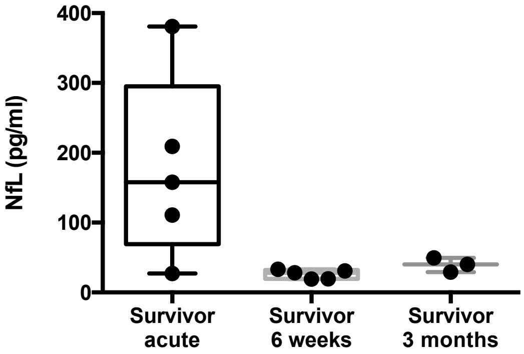

Supplemental Figure 2: Time course of neurofilament light chain serum levels in reversible edema
The NfL serum levels normalize within 6 weeks after reversible edema (n=5).
